# Seroprevalence and associated risk factors for foot and mouth disease virus seropositivity in cattle in selected districts of Gamo zone, Southern Ethiopia

**DOI:** 10.3389/fvets.2022.931643

**Published:** 2022-08-15

**Authors:** Mesfin Shurbe, Bekahegn Simeon, Wasihun Seyoum, Ayelech Muluneh, Ephrem Tora, Edget Abayneh

**Affiliations:** ^1^Department of Animal Science and Health, Livestock and Fishery Research Center, College of Agriculture, Arba Minch University, Arba Minch, Ethiopia; ^2^National Animal Health Diagnostic and Investigation Center, Sebeta, Ethiopia

**Keywords:** foot and mouth disease, Gamo zone, knowledge, perception, seroprevalence

## Abstract

**Background:**

Foot and mouth disease (FMD) is a highly contagious viral disease of cloven-hoofed animals, which hampers livestock production and productivity in Ethiopia. This cross-sectional study was conducted from January to December 2021 to estimate the seroprevalence of FMD in cattle and to assess farmers' knowledge about the disease in selected districts of the Gamo zone. Three districts and two kebeles (smallest administrative division) from each district were purposively sampled using a simple random sampling technique to select individual animals from each kebeles. A total of 384 sera samples were collected, and concurrently, 100 farmers were interviewed. The samples were tested for antibodies against nonstructural proteins of the FMD virus using a 3ABC enzyme-linked immunosorbent assay (ELISA). Univariable and multivariable logistic regressions were used to analyze FMD-associated risk factors.

**Result:**

The questionnaire survey result revealed that among the interviewed farmers, 66% of farmers had knowledge about the disease, and 28% of farmers reported having a case of FMD in at least one cattle in their farm in the previous 6 months. The overall seroprevalence of FMD in cattle was 26.8%. The multivariable logistic regression revealed that age, breed, and agroecology had a significant association with seropositivity. Higher seroprevalence (64.57%) was observed in lowland, followed by midland (9.30%) and highland (5.88%). Study animals from lowland areas were 9.26 times more likely to be seropositive (OR = 9.26, CI = 2.22–38.62) for FMD than highland animals. Also, adult animals were 9.01 times (OR = 9.01, CI = 3.18–25.53) more likely to be seropositive for the disease than young animals. The multivariable logistic regression revealed that crossbreeds have an 84.7% (OR = 0.153, CI = 0.028–0.82) lower likelihood to be seropositive to FMD than local breeds.

**Conclusion:**

This study result confirms that FMD is highly prevalent in the study area, and farmers' knowledge regarding disease transmission and vaccine availability is minimal. Hence the regional concerned bodies should implement FMD vaccination campaigns and create awareness for smallholder farmers regarding the disease transmission, FMD vaccine schedule, and vaccination importance.

## Background

Ethiopia has the largest livestock population in Africa comprising 60.9 million cattle, 31.3 million sheep, and 32.7 million goats. The livestock sector plays a crucial role in the national economy, as well as in the socioeconomic development of millions of rural smallholder farmers; it has considerable prospective opportunities for income generation, employment, and poverty alleviation (1, 2) and sustains livelihoods for 80% of all rural population (3).

However, livestock production in the country is severely affected by several constraints, including the widespread distribution of animal diseases in different agroecological zones, resulting in high annual mortality rates (4). Of the animal diseases hindering productivity, foot-and-mouth disease (FMD) is considered a bottleneck for livestock production and has become the leading cause of blocking the trade of live animals and animal products (5, 6).

Foot and mouth disease is a contagious viral disease caused by the FMD virus (FMDV) of the genus *Aphthovirus*, in the family of Picornaviridae, and it affects cloven-hoofed animals (7). The FMDV genome consists of an 8,400-nucleotide single-stranded ribonucleic acid (ssRNA) that encodes a polypeptide that cleaves into several nonstructural proteins (NSPs) and four structural proteins (SPs) (8). The disease is clinically characterized by fever; loss of appetite; vesicles on the tongue, dental pad, gums, soft palate, nostrils, or muzzle that lead to excess salivation; vesicular eruptions on the feet and teats; and sudden death of young stock (9). There are seven serotypes of FMDVs (i.e., O, A, C, Asia 1, SAT 1, SAT 2, and SAT 3) (10), which have distinct immunologic, antigenic, and genetic properties (11). At present, five serotypes of FMDVs have been reported in Ethiopia (12), which indicates that the disease is endemic in Ethiopia, and varying degrees of their existence were found in different parts of the country, with seroprevalence ranging from 8.18% in South Omo to 44.2% in selected districts of Afar Pastoral Area (13).

The FMD is diagnosed using a combination of history, clinical symptoms, and laboratory investigations. FMDV can be isolated on cell cultures, the viral nonstructural protein can be detected using ELISAs, and the presence of viral genomic material can be detected using PCR assays (14). Anti-NSP antibody testing is commonly utilized to distinguish infected animals from vaccinated animals in both FMD endemic areas (15) and FMD-free countries (16).

The current situation of FMD in Ethiopia is alarming due to its wide distribution with variant strains in different parts of the country. Thus, livestock are at risk from endemic strains as well as from antigenic variants prevailing in neighboring countries (17). Regardless of its wide geographic distribution, broad host range, ability to establish carrier status, and poor cross-immunity, the control of the disease is complicated in FMD endemic areas due to limited disease surveillance together with lack of molecular characterization and lack of proper identification of the origin of the disease (18). Thus, continuous FMD disease surveillance together with serotyping of the virus is a paramount role in undertaking efficient control schemes. Therefore, this study was designed to determine the seroprevalence of antibodies against FMDV and assess the potential risk factors associated with the seroprevalence of the disease in Gamo zone, southern Ethiopia.

## Materials and methods

### Study area description

**Figure 1:**
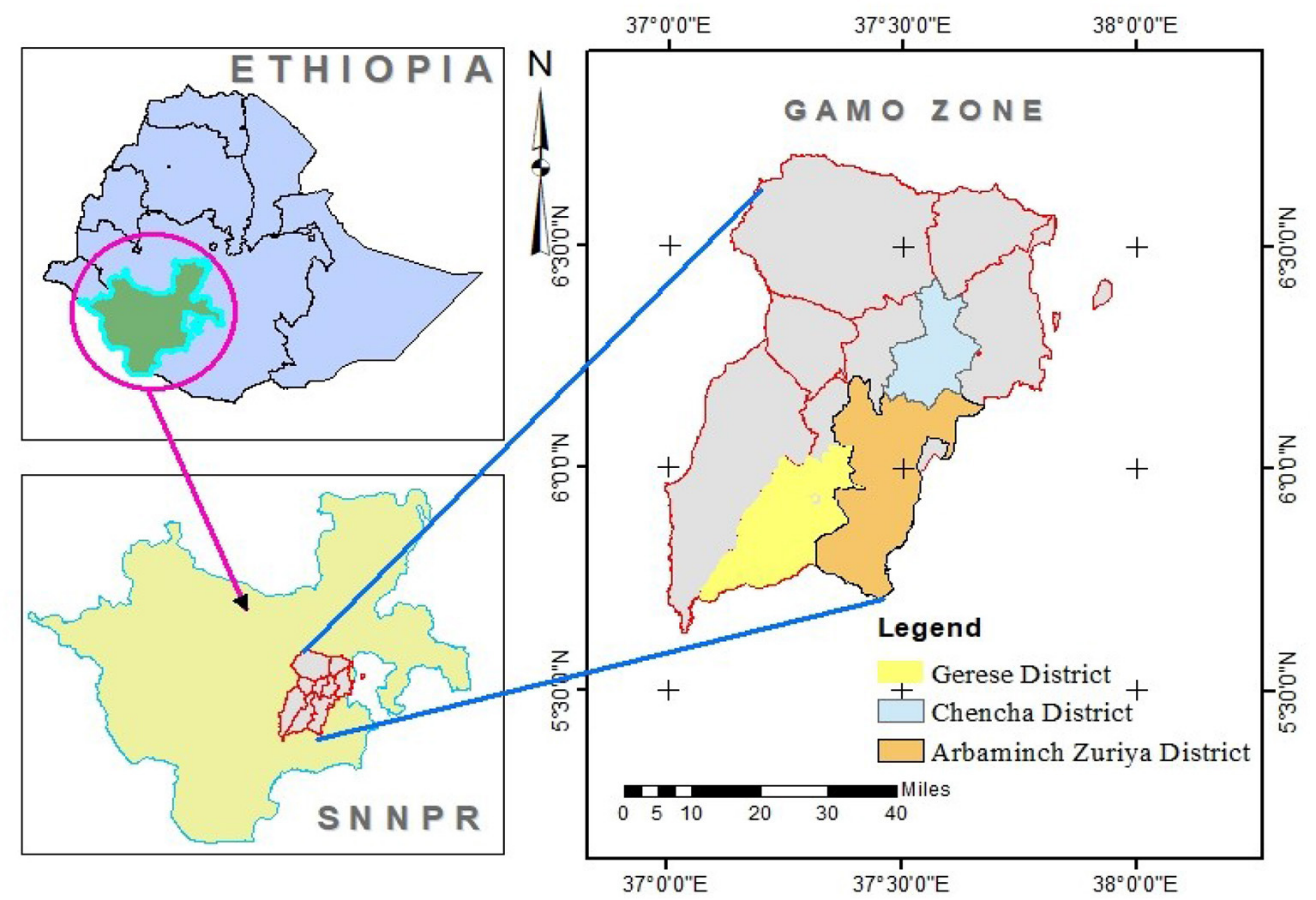
Map of the study area.

This study was conducted from January to December 2021 in three purposely selected districts, namely, Geresse, Arbaminch Zuria, and Chencha of Gamo zone, Southern Ethiopia (Figure 1).

Geresse district is one of the newly established districts of Gamo zone, formerly which is part of Bonke district. Gresse district is located 55 km from the capital of Gamo zone, which is situated between 800 m and 2,700 m above sea level. The district has 23 kebeles, of which 43% kebeles are highland, 32% kebeles are midland, and 25% kebeles are lowland. The total area of the district is estimated to be 66,683.02 hectares with an annual rainfall of 800–1,200 mm. The estimated livestock populations of the district are 137,171 cattle, 189,557 sheep, 65,758 goats, 36,566 equines, and 226,026 poultry (19).

Arbaminch Zuria district has a bimodal rainfall system, short rain season that occurs from January to April and long rain season that occurs from June to September. The altitude of the district ranges from 1,001 to 2,500 m above sea level. The district has two agroecological zones, namely, Woina Dega (midland) and Kola (lowland). The district has 18 kebeles, of which 8 kebeles were found in midland and the remaining 10 kebeles were found in lowland agroecological zones of the district. Within the district, livestock husbandry is generally characterized by an extensive farming system, in which animals are allowed to graze freely during day time and kept in open enclosures during night time. The livestock populations of the district are 101,628 cattle, 27,339 sheep, 42,662 goats, 3,204 equines, and 140,050 poultry (20).

Chencha District is situated between 1,300 m and 3,250 m above sea level. Astronomical location of Chencha Woreda is between 37 29′ 57″ East to 37 39′ 36″ West and between 6^0^ 8′ 55″ North and 6^0^ 25′ 30” South. Due to a high altitudinal range, the area is characterized by diverse agroclimatic distribution. The district is divided into two agroecological zones, namely, Dega and Weyna Dega, which account for about 82 and 18% of the total area, respectively. The rainfall regime in the district is bimodal. The first round of rain occurs between March to April. The second round of rain occurs from June to August. The annual rainfall distribution in the district varies between 900 mm to 1,200 mm. The minimum and maximum temperatures in the district range from 11°C to 13°C and 18°C to 23°C, respectively. The farming system in the district is a mixed farming system where the crop sub-system and the livestock sub-system are practiced. Chencha has 67,269 cattle, 106,594 sheep, 11,870 goats, and 22,554 equines (21).

### Study design

A cross-sectional study was conducted from January to December 2021 to estimate the seroprevalence and associated risk factors of FMD. In addition, a survey was used to assess farmers' knowledge of FMD in the study area.

### Study population

The study animals were cattle that were kept under different management systems (extensive, intensive, and semi-intensive farming systems). All local breed and crossbreed cattle that were > 6 months of age were included in the study. In the sample collection period, information concerning animal level risk factors such as age, sex, and breed was collected and recorded. The age of each study animal was determined by consulting the owners of the cattle. Accordingly, animals were categorized as calves (<2 years), young (2–4 years), and adults (> 4 years) (22).

### Inclusion criteria

All local breed and crossbreed cattle > 6 months of age were included in the study. Also, cattle owners who showed willingness to participate in the survey were included in the study.

### Sampling method and sample size determination

Three districts, namely, Geresse, Chencha, and Arbaminch Zuria, and two kebeles from each district were selected purposively based on their agroecology, proximity to livestock market, contact with wildlife, accessibility for transportation and immediate laboratory procedure, and population density. Then, simple random sampling was employed to select each study animal from each kebeles. The sample size required for the study was calculated based on the following formula (23):


$$\begin{array}{l} \text{n = 1}{\text{.96}}^{\text{2}}{\text{ × P}}_{\text{exp}}{\text{ × (1 - P}}_{\text{exp}}\text{) }\\ {\text{ d}}^{\text{2}} \end{array}$$

where *n* = sample size, P_exp_ = expected prevalence, and *d* = absolute precision.

Considering the expected prevalence of 50% with 95% confidence level and 5% absolute precision, the sample size computed was 384. Then, proportionate numbers of animals were sampled from each of the three districts based on their cattle population size. Consequently, 172, 127, and 85 animals were sampled from Geresse, Arbaminch Zuria, and Chencha districts, respectively.

The survey of farmers' knowledge was carried out in three districts in conjunction with blood sample collection. From cattle owners whose cattle were sampled for serology, a total of 100 farmers were randomly selected from the three districts. Accordingly, 33 individuals from Geresse, 34 individuals from Arbaminch Zuria, and 33 individuals from Chencha districts were interviewed. The sample size was determined using the formula (*n* = 0.25/SE^2^) as per Arsham (24) at the standard error (SE) of 0.05 with 95% confidence interval.

## Study methodology

### Questionnaire survey

A semi-structured questionnaire was used to assess the farmers' knowledge of FMD. The questionnaire was pretested and modified before the final interviews were conducted. The questionnaire was designed to assess whether the informants know FMD, its clinical signs, source of infection, and prevention and control methods.

### Blood sample collection

From each animal, 10 ml of blood was collected from the jugular vein using a 21-gauge needle, and serum samples were transported in a cold chain to the National Animal Health Diagnostic and Investigation Center (NAHDIC) and stored at −20°C until further use (25).

### Serological diagnostic tests

The collected sera were tested by FMDV 3ABC-Ab ELISA (ID Screen® FMD NSP Competition, ID-VET, Grabels, France) at the NAHDIC according to the manufacturers' recommendation and the procedure provided by the OIE Manual of Diagnostic Tests and Vaccines for Terrestrial Animals (14). Percentage inhibition equal to or <50% was considered positive.

### Associated risk factors

Age, sex, body condition, and breed of study animals were considered as intrinsic risk factors of FMD during the study period, while management system (extensive, semi-intensive, and intensive), herd composition, herd size, history of movement of animals, contact with wildlife, awareness of farmers, agroecology, and communal grazing and watering practices were considered as extrinsic risk factors for FMD. This information was recorded in the prepared data sheet for each animal. Herd size is classified into *three* categories such as small herd <10 animals, medium herd 10–50 animals, and large herd >50 animals (26).

### Data management and analysis

Data generated by laboratory investigations and the questionnaire survey were recorded and coded using a Microsoft Excel spreadsheet (Microsoft Corporation) and was analyzed using STATA version 14.0 for Windows (Stata Corp. College Station, TX, USA). Descriptive statistics were used to present the survey results and to calculate the proportion of FMD-related risk factors. Disease seroprevalence was computed by dividing the number of positive ELISA results by the total number of collected samples. In addition, univariate and multivariate logistic regression analyses were conducted to identify the main FMD risk factors, including sex, age, breed, body condition score, agroecology, herd size, herd composition, management system, communal grazing, communal watering, and cattle owner awareness of the FMD symptoms and risks. After checking the data for collinearity, all variables with *p* < 0.25 in the univariable analysis were subjected to stepwise backward multivariable logistic regression analysis. Hosmer-Lemeshow test was performed to check the goodness of fit of the final model. In the serological study, odds ratios (ORs) were calculated to determine the degree of association between each risk factor and FMD seropositivity. In all analyses, a 95% confidence interval (CI) was calculated, and *p* < 0.05 was considered statistically significant.

## Results

### Cattle owners' knowledge of FMD

The survey revealed that 66% (66/100) of the surveyed farmers were aware of the FMD and were familiar with its local name “Massa.” Those who knew about the disease were instructed to indicate the typical symptoms of the disease. The most commonly cited symptoms were hyper-salivation lesions on the mouth (37.8%, 25/66) and feet (35%, 23/66), lameness (24.2%, 16/66), and inappetence (3.2%, 2/66). In addition, 44% (29/66) of the farmers that were aware of FMD also indicated that they were familiar with the possible causes of the disease. About 30% (20/66) of the farmers believed that contact with infected animals during grazing led to FMD, and 14% (9/66) ascribed the disease outbreaks to the introduction of diseased animals into a herd. However, 98% (98/100) of the interviewed cattle owners did not know about a vaccine that would protect their livestock from FMD.

### Foot and mouth disease: 6-month occurrence

The survey results revealed that 28% (28/100) of the surveyed cattle owners reported having a case of FMD in at least one cattle in their farm in the previous 6 months. Those that reported FMD cases were instructed to indicate the disease management strategy they had adopted. While 50% (14/28) of the farmers who had FMD cases in their livestock opted for medical treatment, 32% (9/28), 7% (2/28), 7% (2/28), and 4% (1/28) of this subgroup chose isolation, selling, slaughtering, and doing nothing, respectively. Cattle owners' questionnaire responses are summarized in Table 1.

**Table 1:** Cattle owners' response to knowledge, prevention, and control practices of FMD.

**Variables**	**Number of respondents**	**Response (%)**
FMD knowledge		
Yes	66	66
No	44	44
FMD vaccine information		
Yes	1	1
No	99	99
Symptoms		
Hypersalivation	25	37.8
Lesions on feet and mouth	23	34.8
Lameness	16	24.2
Inappetence	2	3.2
FMD 6 months occurrence		
Yes	28	28
No	72	72
Control method		
Treatment	14	50
Isolation	9	32
Selling	2	7
Slaughtering	2	7
Doing nothing	1	4
Knowledge about causes of FMD		
Contact with wild life	0	0
Contact with infected animal	20	30
Introduction of infected animal	9	14
Do not know	37	56

### Overall seroprevalence of foot-and-mouth disease virus

The study revealed that out of 384 samples tested, 103 (26.82%) samples were positive for the presence of antibodies against FMDV NSP (Table 2).

**Table 2:** Summary of the risk factors of FMD.

**Variable**	**Categories**	**No. of examined**	**No. of positive**	**Prevalence**
Age	Calves	102	5	4.90
	Young	113	30	26.55
	Adult	169	68	40.24
Sex	Male	89	25	28.09
	Female	295	78	26.44
Breed	Local	295	101	34.24
	Cross	89	2	2.25
Body Condition	Poor	70	16	22.86
	Medium	169	45	26.63
	Good	145	42	28.97
Agroecology	Lowland	127	5	5.88
	Midland	172	16	9.30
	Highland	85	82	64.57
Management	Intensive	75	5	6.67
system	Semi intensive	221	61	27.60
	Extensive	88	37	42.05
Herd size	Small	242	30	12.4
	Medium	58	17	29.31
	Large	84	56	66.67
Herd composition	Mixed	246	26	18.84
	Not mixed	138	77	31.30
Contact with	Yes	177	19	9.18
wild life	No	207	84	47.46
Communal grazing	Yes	273	8	7.21
	No	111	95	34.80
Movement history	Yes	167	67	40.12
	No	217	36	16.59

### Association of risk factors with seropositivity of FMD

The association between seropositivity and hypothesized risk factors was analyzed using both univariable and multivariable logistic regressions. From a total of 10 hypothesized risk factors that were statistically significant when analyzed by univariable logistic analysis, only 3 risk factors had a statistically significant (*p* < 0.05) association with seroprevalence of FMD in a final model. The result of the two models is summarized and presented in Tables 3, 4.

**Table 3:** Univariable logistic regression results of risk factor analysis.

**Variable**	**OR**	**CI**	***p*-value**
Sex			
Female	-	-	-
Male	1.08	0.64–1.85	0.758
Age			
Calves	-	-	-
Young	7.01	2.60–18.89	0.001
Adult	13.06	5.05–33.77	0.001
Breed			
Local	-	-	-
Cross	0.044	0.011–0.183	0.001
Body condition			
Poor			
Medium	1.22	0.64–2.35	0.543
Good	1.37	0.701–2.67	0.345
Agroecology			
Highland	-	-	-
Midland	1.64	0.58–4.64	0.350
Lowland	29.15	11.01–77.21	0.000
Herd size			
Small	-	-	-
Medium	2.93	1.48–5.79	0.002
Large	14.13	7.81–25.57	0.001
Herd composition			
Not mixed			
Mixed	1.96	1.185–3.250	0.009
Management type			
Intensive			
Semi-intensive	5.34	2.06–13.85	0.001
Extensive	10.15	3.73–27.64	0.001
Communal Grazing			
No			
Yes	6.87	3.20–14.71	0.001
Movement History			
No			
Yes	3.36	2.09–5.41	0.001
Contact with wildlife			
No	-	-	-
Yes	8.93	5.12–15.59	0.000

CI, Confidence interval; OR, Odds ratio.

**Table 4:** Multivariable logistic regression results of risk factor analysis.

**Variable**	**OR**	**CI**	***p*-value**
Age			
Calves	-	-	-
Young	4.55	1.50–13.67	0.007
Adult	9.01	3.18–25.53	0.001
Breed			
Local	-	-	-
Cross	0.153	0.028–0.82	0.029
Agroecology			
High land	-	-	-
Mid land	0.725	0.21–2.55	0.617
Low land	9.26	2.22–38.62	0.002

*Result of the Hosmer-Lemeshow goodness-of-fit test χ^2^ = 9.41; p = 0.309.

The age of the study population was categorized into three groups, namely, calves (6 months to 2 years), young (2–4 years), and adult (> 4 years). Higher seroprevalence of FMD was seen in adult animals (40.24%), followed by young (26.55%) and calves (4.90%). The multivariable logistic regression result revealed that adult animals were 9.01 times (CI = 3.18–25.53) more likely to be positive for the disease than young animals.

The other hypothesized intrinsic factor for FMD was breed, which was categorized as local breed and crossbreed. The prevalence of FMD is higher in local breeds (34.24%) than crossbreeds (2.25%). The multivariable logistic regression revealed that the odds of being seropositive is 84.7% (OR = 0.153, CI = 0.028–0.82) less likely in local breeds than crossbreeds.

The association between seropositivity and hypothesized extrinsic risk factor like agroecology was analyzed using multivariable logistic regression and is summarized in Table 4. Study animals that were living in lowland areas were 9.26 times more likely to be seropositive (OR = 9.26, CI = 2.22–38.62) for FMD than study animals from highland areas. The highest seroprevalence (64.57%) was observed in the lowland district followed by the midland (9.30%) and highland (5.88%) districts.

## Discussion

### Farmer's knowledge and perception on FMD

This study revealed that 66% of the respondents had knowledge regarding FMD clinical signs with its local name “Massa.” This finding goes in line with a study conducted in the Amhara region by Mesfine et al. (27), who reported that 82.4% of respondents knew FMD. Also, Tesfaye et al. (28) reported that pastoralists living at the Borena zone are well aware of the clinical signs of FMD and it was known by the local name Oyale. About 30% (20/66) of the farmers believed that contact with infected animals during grazing led to FMD, and 14% (9/66) ascribed the disease outbreaks to the introduction of diseased animals into a herd. This result strongly agrees with a study conducted in the Amhara region by Mesfine et al. (27), who showed that about 78% of farmers surveyed expect FMD to be transmitted by coming into contact with infected animals during communal grazing and watering activities, and about 22% think that it is primarily by infected animals coming from markets. The survey includes a question about the typical symptoms of the disease for those who have knowledge of it. The most commonly mentioned sign was hypersalivation (37%) followed by lesion on feet and mouth (35%), lameness (24%), and inappetence (4%). This finding goes in line with a study conducted in the Amhara region by Mesfine et al. (27) and in the Oromia region by Bayissa et al. (29), who reported that most of the farmers in the study areas were able to describe clinical signs of FMD. Additionally, the study conducted in the Adea Berga district of central Oromia by Urge et al. (30) showed profuse salivation as the most frequently observed clinical sign (39%), followed by oral cavity and interdigital vesicle (22.6%), lameness (7.5%), and inappetence (7.5%).

The survey results revealed that 28% (28/100) of the surveyed cattle owners reported having a case of FMD in at least one cattle in their farm in the previous 6 months. This finding agrees with a study conducted in Kenya by Nyaguthii et al. (31), who reported that out of a total of 220 smallholder farmers, 13 (5.9%) respondents replied having FMD in at least one cattle in their herd in the previous 6 months. Those that reported FMD cases were instructed to indicate the disease management strategy they had adopted. While 50% (14/28) of the farmers that had FMD cases in their livestock opted for medical treatment, and 32% (9/28), 7% (2/28), 7% (2/28), and 4% (1/28) of this subgroup chose isolation, selling, slaughtering, and doing nothing, respectively. This finding agrees with a study conducted in the Amhara region by Mesfine et al. (27), who reported that about 48% of farmers practiced one or more types of FMD control measures following disease occurrence.

### Seroprevalence and associated risk factors of FMD

The overall prevalence in this study was 26.82%, in agreement with those of other studies in the country (32) and (33) whose overall prevalence was 24.22 and 26.5%, respectively. In the central Tigray zone (34) and the South Omo zone (35), seroprevalences of 26.6% and 23.9% were recorded, respectively. The highest overall seroprevalence reports were in Adiss Abeba (72.1%) (36), followed by 49.2% in Oromiya (30), 41.5% in Tigray's Eastern zone (34), and 40.4% in West Shewa Zone (37). In Southern Ethiopia, the lowest seroprevalence was reported at 9.5%, and in the Gamo Gofa zone and Sidama zone, the seroprevalence input was 6.9 and 5.9%, respectively (35). Those prevalence differences might have emerged from differences in sampling method, study design, and the presence and absence of extrinsic risk factors like agroecology, contact of animals with wildlife, free movement of animals, communal grazing, and communal watering.

In this study, prevalence varied between age groups in a statistically significant manner. This finding is in line with the findings of Dubie and Negash (38), who found a higher prevalence in adult animals than in young animals in a study conducted at the Afar region. Other scholars who reported the same finding were Awel and Dilba (36) in Addis Ababa, Megerssa et al. (35) in Southern Ethiopia, Sulayeman et al. (32) in central Ethiopia, Gelana (39) in Western Oromiya, and Abunna et al. (40) in Dire Dawa. These statistically significant prevalence differences between different age groups reported might be due to increased exposure to disease risk factors as an animal's age increases. In this study area, calves were kept in barns until they were old enough to graze communally. This habit decreases their exposure to the disease. Additionally, calves <1 year are protected from the disease due to their passive maternal immunity (28). In contradiction with the above findings, Gelaye et al. (17) in the Benchi Maji zone and Belina et al. (41) in the Eastern Showa zone reported no statistically significant difference in the seroprevalence of FMD in different age groups.

In terms of breed, the current study has shown a statistically significant difference between local breed and crossbreed animals. This finding agrees with Sulayeman et al. (32), Urge et al. (30), and Ahmed et al. (37) who reported statistically significant differences between the local breed and crossbreed prevalence estimates in central Ethiopia, Welmera district of Oromia region, and West Showa zone, respectively. This study finds a higher prevalence in local breeds than crossbreeds as opposed to Sulayeman et al. (32), Urge et al. (30), and Ahmed et al. (37). Possibly, this result variation was caused by non-proportionate sample allocation, and local breeds were more prone to FMD risk factors such as wildlife contact, free movement, semi-intensive/extensive management systems, and communal grazing. Even though the difference was not statistically significant, Awel and Dilba (36) reported a higher prevalence in local breeds than crossbreeds.

In this study, agroecology displayed statistically significant variations in seroprevalence. The magnitude of seroprevalence decreases when agroecology changes from lowland to midland and from midland to highland. The logistic regression result showed that lowland areas were 29.15 times more likely to be seropositive (OR= 29.15, CI =11.01, 77.21) than highland areas. There is strong agreement with the findings of Megerssa et al. (35) and Mesfine et al. (27), who reported that animals found in midlands and highlands were 83% (OR = 0.17, CI = 0.04–0.85) less likely to be seropositive for FMD than lowland animals. Similarly, Tesfaye et al. (42) found significant differences between areas of different altitudes with a prevalence of 53.6% and 10.1% at low and high altitudes, respectively. This prevalence variation arises due to an increase in exposure of animals to the putative risk factors in lowland areas.

## Conclusion and recommendations

The study revealed that more than half of the respondents were aware of FMD but had limited knowledge about the presence of the FMD vaccine. Moreover, the survey also revealed that farmers' awareness of the source of FMD infection was minimal. Instead, they followed a strategy of selling, slaughtering, isolating, and doing nothing as a means of preventing it. The serological findings confirmed that the disease is endemic in this study area. An analysis of multivariable logistic regression showed that age, breed, and agroecology are statistically significant risk factors for the disease. The seropositivity of the disease is higher in animals that are living in lowland areas than in midland and highland areas. Therefore, the regional government should give an emphasis on massive vaccination campaigns, especially for animals found in lowland areas, and create awareness through training of smallholder farmers about the disease transmission, FMD vaccination schedule, and vaccine importance.

## Data availability statement

The datasets presented in this study can be found in online repositories. The names of the repository/repositories and accession number(s) can be found at: https://zenodo.org/record/5834604#.YmuF1tPMJPY.

## Ethics statement

The animal study was reviewed and approved by Arba Minch University Animal Ethical Clearance Committee. Written informed consent was obtained from the owners for the participation of their animals in this study.

## Author contributions

MS, BS, and WS participated in the study design, data analysis, write-up of the draft, and final version of the manuscript. AM, ET, and EA participated in the data collection through questionnaire and encoding of raw data. All authors contributed to the article and approved the submitted version.

## Funding

This work was funded by Arba Minch University with funding grant number GOV/AMU/LFRC/TH6/01/13 and with reference number AMU-9/37/100.

## Conflict of interest

The authors declare that the research was conducted in the absence of any commercial or financial relationships that could be construed as a potential conflict of interest.

## Publisher's note

All claims expressed in this article are solely those of the authors and do not necessarily represent those of their affiliated organizations, or those of the publisher, the editors and the reviewers. Any product that may be evaluated in this article, or claim that may be made by its manufacturer, is not guaranteed or endorsed by the publisher.

## References

[1] Central Statistical Agency (CSA). Report on livestock and livestock characteristics, volume II, Addis Ababa. Statistical Bulletin. (2018) 2:587.

[2] Food Agriculture Organization of the United Nations. Supporting Low Emissions Development in the Ethiopian Dairy Cattle Sector. Rome: Food and Agriculture Organization of the United Nations (2017). Available online at: https://www.google.com/url?sa=t&rct=j&q=&esrc=s&source=web&cd=&cad=rja&uact=8&ved=2ahUKEwjnwLHU5q35AhWP-aQKHVmYD3EQFnoECBUQAQ&url=http%3A%2F%2Fwww.fao.org%2F3%2Fa-i6821e.pdf&usg=AOvVaw2i7ASESLjtwUHFrZDCt9fj

[3] Behnke R,. Contribution of Livestock to the Economies of IGAD Member States: Study Findings, Application of the Methodology in Ethiopia Recommendations for 69 Further Work. IGAD LPI Working Paper 02-10. Odessa Centre, IGAD Livestock Policy Initiative, Great Wolford, UK. (2010). Available online at: https://core.ac.uk

[4] Ganeshkumar B. Economic impact of foot-and-mouth disease in India, scientific developments and technical challenges in the progressivecontrol of foot-and-mouth disease in South Asia, New Delhi. India Glob Res Alli. (2012) 13:5.

[5] Knight-Jones T Rushton J. The economic impacts of foot and mouth disease–What are they, how big are they and where do they occur? Prev Vet Med. (2013) 112:161–73. 10.1016/j.prevetmed.2013.07.013 23958457 PMC3989032

[6] Mansley LM Donaldson AI Thrusfield MV. Destructive tension: mathematics versus experience – the progress and control of the foot and mouth disease epidemic. Rev Sci-entifiqueet Tech. (2011) 30:483–98. 10.20506/rst.30.2.2054 21961220

[7] Quinn PJ Markey BK Leonard FC Hartigan P Fanning S Fitzpatrick E. Veterinary Microbiology and Microbial Disease. John Wiley and Sons (2011).

[8] Jamal S Belsham GJ. Foot and mouth disease: past, present and future. Vet Res. (2013) 44:116. 10.1186/1297-9716-44-116 24308718 PMC4028749

[9] Quinn PJ Markey BK Carter ME Donnelly WJ Leonard FC. Veterinary Microbiology and Microbial Disease. Blackwell Science Ltd, A Blackwell publishing company (2005). p. 402–7

[10] Tekleghiorghis T Weerdmeester K van Hemert-Kluitenberg F Moormann RJ Dekker A. Comparison of test methodologies for foot-and-mouth diseasevirus serotype A vaccine matching. Clin Vaccine Immunol. (2014) 21:674–83. 10.1128/cvi.00034-14 24623625 PMC4018896

[11] Rufael T Catley A Bogale A Sahle M Shiferaw Y. Foot and mouth disease in the Borana pastoral system, southern Ethiopia and implications for livelihoods and international trade. Trop Anim Health Prod. (2008) 40:29–38. 10.1007/s11250-007-9049-6 18551776

[12] Abdela N. Sero-prevalence, risk factors and distribution of foot and mouth disease in Ethiopia. Acta Tropica. (2017) 169:125–32. 10.1016/j.actatropica.2017.02.01 28209551

[13] Jenbere S Etana M Negussie H. Study on the risk factors of footand mouth disease in selected districts of Afar Pastoral Area, NortheastEthiopia. J Anim Vet Adv. (2011) 10:1368–72. 10.3923/javaa.2011.1368.1372

[14] Office of International des Epizootics (OIE). Foot and Mouth Disease, Manual of Diagnostic Tests and Vaccines For Terrestrial Animals (Mammals, Birds and Bees) 7th ed, vol 1. Paris (2012). p. 145–73.

[15] Brocchi E Bergmann I Dekker A Paton D Sammin D Greiner M. Comparative evaluation of six ELISAs for the detection of antibodies to the non-structural proteins of foot-and-mouth disease virus. Vaccine. (2006) 24:6966–79. 10.1016/j.vaccine.2006.04.050 16753241

[16] Barnett P Geale D Clarke G Davis J Kasari T. A review of OIE country status recovery using vaccinate-to-live versus vaccinate-to-die foot-and-mouth disease response policies I: benefits of higher potency vaccines and associated NSP DIVA test systems in post-outbreak surveillance. Trans Bound Emerg Dis. (2015) 62:367–38. 10.1111/tbed.12166 24112127

[17] Gelaye E Ayelet G Abera T Asmare K. Seroprevalence of foot and mouth disease in Bench Maji zone, Southwestern Ethiopia. Inernational J Biochem Biophys Mol Biol. (2009) 1:005010.

[18] MacLachlan NJ Dubovi EJ Barthold SW Swayne DE Winton JR. Fenner's Veterinary Virology. California, CA: Elsevier Academic Press (2016) 426–40.

[19] Geresse District Livestock and Fishery Resource Office (GDLFRO). Report from Arba Minch Zuria District Livestock and Fishery Resource Office, Gamo Zone, SNNPR (Unpublished report). (2020).

[20] Arbaminch Zuria District Livestock and Fishery Resource Office (AZDLFRO). Report from Arbaminch Zuria District Livestock and Fishery Resource Office, Gamo Zone, SNNPR (Unpublished report). (2020).

[21] Chencha District Livestock and Fishery Resource Office (CDLFRO). Report from Chencha District Livestock and Fishery Resource Office, Gamo Zone, SNNPR (Unpublished report). (2020).

[22] Abdulahi M Esayas T Hailu D. Seroprevalence of bovine foot and mouth disease (FMD) in Awbere and Babille districts of Jijiga zone, Somalia Regional State, Eastern Ethiopia: Afr. J Microbiol Res. (2011) 5:3559–63. 10.5897/AJMR11.750

[23] Thrusfield M Christley R Brown H Diggle P French F Howe K. Veterinary Epidemiology. 4th ed. Royal School of Veterinary Studies University of Edinburgh: Willey Blackwell (2018). p. 276. 10.1002/9781118280249

[24] Arsham H,. Descriptive Sampling Data Analysis. Statistical Thinking for Managerial Decision Making (2002). Available online at: http://home.ubalt.edu/ntsbarsh/Business-stat/opre504.htm

[25] Office of International des Epizootics (OIE). Foot and Mouth Disease (FMD). Paris, France (2019).

[26] Megersa M FeyissaA Wondimu A Jibat T. ‘Herd composition and characteristics of dairy production in Bishoftu Town, Ethiopia’. J Agric Ext Rural Dev. (2011) 3:113–7.

[27] Mesfine M Nigatu S Belayneh N Jemberu WT. Sero-Epidemiology of foot and mouth disease in domestic ruminants in Amhara Region. Front Vet Sci. (2019) 6:130. 10.3389/fvets.2019.00130 31114792 PMC6503644

[28] Tesfaye A Sehale M Abebe A Muluneh A. Sero-prevalence of foot and mouth disease in cattle in Borena Zone, Oromia regional state, Ethiopia. Ethiop Vet. J. (2016) 20:55–662. 10.4314/evj.v20i1.4

[29] Bayissa B Ayelet G Kyule M Jibril Y Gelaye E. Study on seroprevalence, riskfactors, and economic impact of foot-and-mouth disease in Borena pastoraland agro-pastoral system, southern Ethiopia. Trop Anim Health Prod. (2011) 43:759–66. 10.1007/s11250-010-9728-6 21249448

[30] Urge B Dawo F Alemu Z Senbeta B Aliyi A Muluneh A. Foot and mouth disease virus infection seroprevalence study in dairy cattle reared by smallholder farmers in Welmera District, Central, Oromiya Ethiopia. J Vet Heal Sci. (2020) 1:5–9. 10.33140/JVHS.01.01.03

[31] Nyaguthii DM Armson B Kitala PM Sanz-Bernardo B Di Nardo A Lyons NA. Knowledge and risk fac-tors for foot-and-mouth disease among small-scale dairy farmers in an endemic setting. Vet Res. (2019) 50:33. 10.1186/s13567-019-0652-0 31088554 PMC6518695

[32] Sulayeman M Dawo F Mammo B Gizaw D Shegu D. Isolation, molecular characterization and sero-prevalence study of foot-and-mouth disease virus circulating in central Ethiopia. BMC Vet Res. (2018) 14:1–10. 10.1186/s12917-018-1429-9 29587741 PMC5870258

[33] Sahle MH Venter PE. An epidemiological study on the genetic relationships of foot and mouth disease viruses in east Africa (PhD dissertation). South Africa: University of Pretoria (2004). p. 110

[34] Ayelet G Gelaye E Negussie H Asmare K. Study on the epidemiology of foot and mouth disease in Ethiopia. OIE Revue Scientifique et Technique. (2012) 31:789–798. 10.20506/rst.31.3.2153 23520733

[35] Megersa B Beyene D Abunna F Regassa A Amenu K Rufael T. Risk factors for foot and mouth disease seroprevalence in indigenous cattle in Southern Ethiopia: the effect of production system. Trop Anim Health Prod. (2009) 41:891–8. 10.1007/s11250-008-9276-5 19052894

[36] Awel SM Dilba GM. Seroprevalence and molecular detection of foot and mouth disease virus in dairy cattle around Addis Ababa, Central Ethiopia. Vet Med Res Rep. (2021) 12:187–97. 10.2147/VMRR.S317103 34285888 PMC8285296

[37] Ahmed B Megersa L Mulatu G Siraj M Boneya G. Seroprevalence and associated risk factors of foot and mouth disease in cattle in West Shewa Zone, Ethiopia. Hindawi Veterinary Medicine International (2020) 2020:6821809. 10.1155/2020/6821809 32292580 PMC7150713

[38] Dubie N Negash W. Seroprevalence of bovine foot and mouth disease (FMD) and its associated risk factors in selected districts of Afar region, Ethiopia. Vet Med Sci. (2021) 7:1678–87. 10.1002/vms3.574 34273244 PMC8464255

[39] Gelana M. Sero-prevalence study on foot and mouth disease in selected districts of Western Oromia. J Pharmacy Alternat Med. (2016) 13:15–8. Available online at: https://core.ac.uk/download/pdf/234689568.pdf

[40] Abunna F Fikru S Rufael T. Sero-prevalence of Foot and Mouth Disease (FMD) at Dire Dawa and Its Surroundings, Eastern Ethiopia. Global Veterinaria. (2013) 11:575–8. 10.5829/idosi.gv.2013.11.5.8113

[41] Belina D Girma B Mengistu S. Sero-Prevalence of Bovine Foot and Mouth Disease in Selected Districts of Eastern Showa Zone, Oromia Regional State. Ethiopia. (2016) 16:6–7.

[42] Tesfaye A Mengistu A Rufael T. ‘Sero-prevalence status of foot and mouth disease in the North Western Amhara Regional State. Ethiopia. (2016) 20:43–53. 10.4314/evj.v20i2.4

